# Transcriptome dynamics of the microRNA inhibition response

**DOI:** 10.1093/nar/gkw091

**Published:** 2016-02-09

**Authors:** Jiayu Wen, Eleonora Leucci, Roberto Vendramin, Sakari Kauppinen, Anders H. Lund, Anders Krogh, Brian J. Parker

**Affiliations:** 1 The Bioinformatics Centre, Department of Biology, University of Copenhagen, Ole Maaloes Vej 5, 2200 Copenhagen N, Denmark; 2 Biotech Research and Innovation Centre (BRIC), University of Copenhagen, Ole Maaloes Vej 5, 2200 Copenhagen N, Denmark; 3 Laboratory for Molecular Cancer Biology, Center for the Biology of Disease, VIB, 3000 Leuven, Belgium; Laboratory for Molecular Cancer Biology, Center of Human Genetics, VIB, 3000 Leuven, Belgium; 4 Department of Haematology, Aalborg University Hospital, A.C. Meyers Vnge 15, 2450 Copenhagen SV, Denmark; 5 Bioinformatics Institute, Agency for Science, Technology and Research (A*STAR), 30 Biopolis street, #07-01, Singapore 138671

*Nucl. Acids Res*. 43 (13): 6207–6221. doi: 10.1093/nar/gkv603

The authors wish to correct the full name of Elenora Leucci to Eleonora Leucci.

